# Supplementary figures and images for: Linking multiple serological assays to infer dengue virus infections from paired samples using mixture models
Source: PLoS Comput Biol. 2025 Nov 25;21(11):e1013708. doi: 10.1371/journal.pcbi.1013708 (PMC12646409; doi:10.1371/journal.pcbi.1013708)

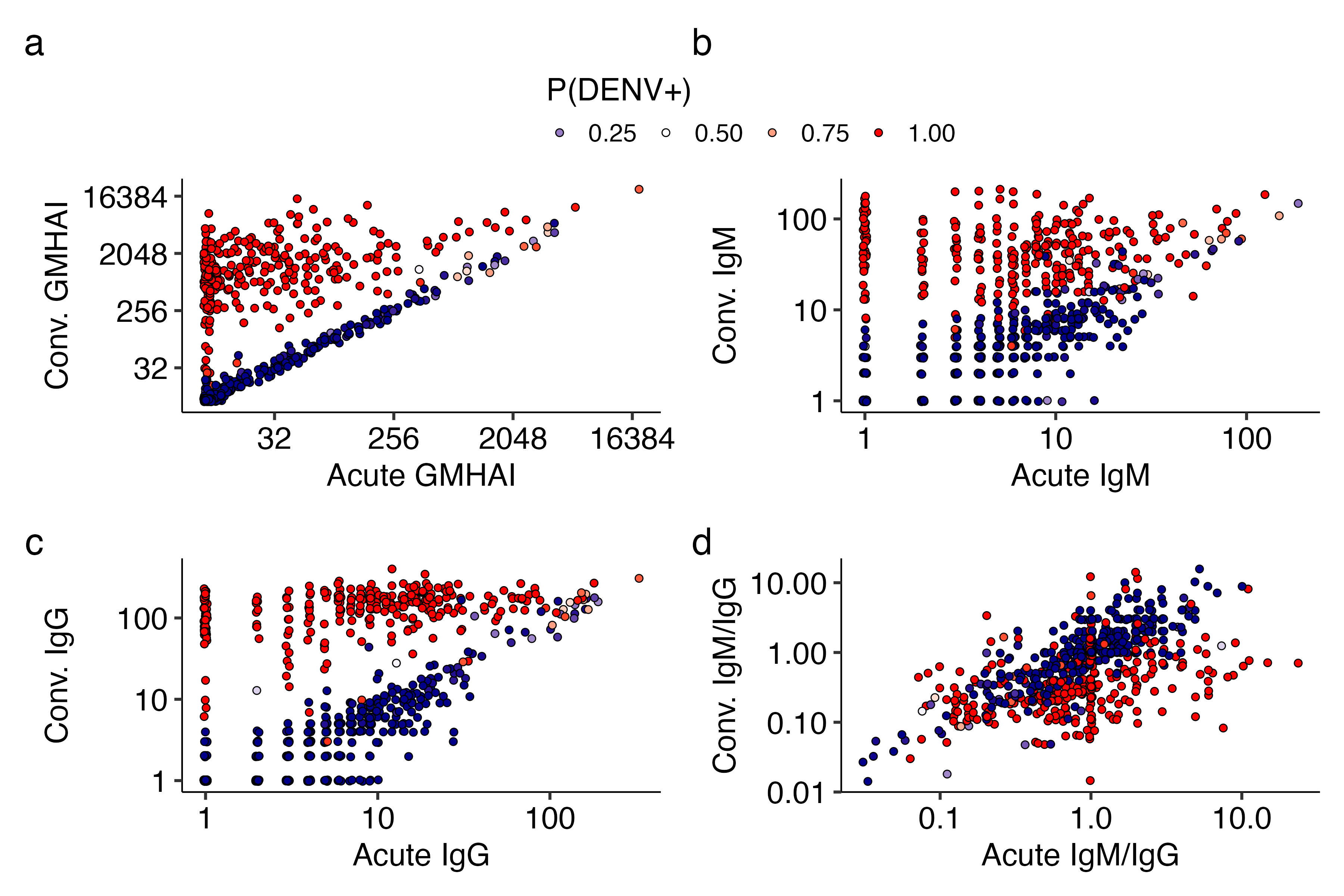

Supplement: S1 Fig — infection found during model testing for a model that accounts for time since symptom onset. a) Geometric mean titers of a haemagglutination inhibition assay (GM HAI) for all four serotypes of dengue virus. b) Immunoglobulin G (IgG) c) Immunoglobulin M (IgM) d) Ratio of IgM to IgG at both acute and convalescent sera samples. (TIF) [file pcbi.1013708.s001.tif]

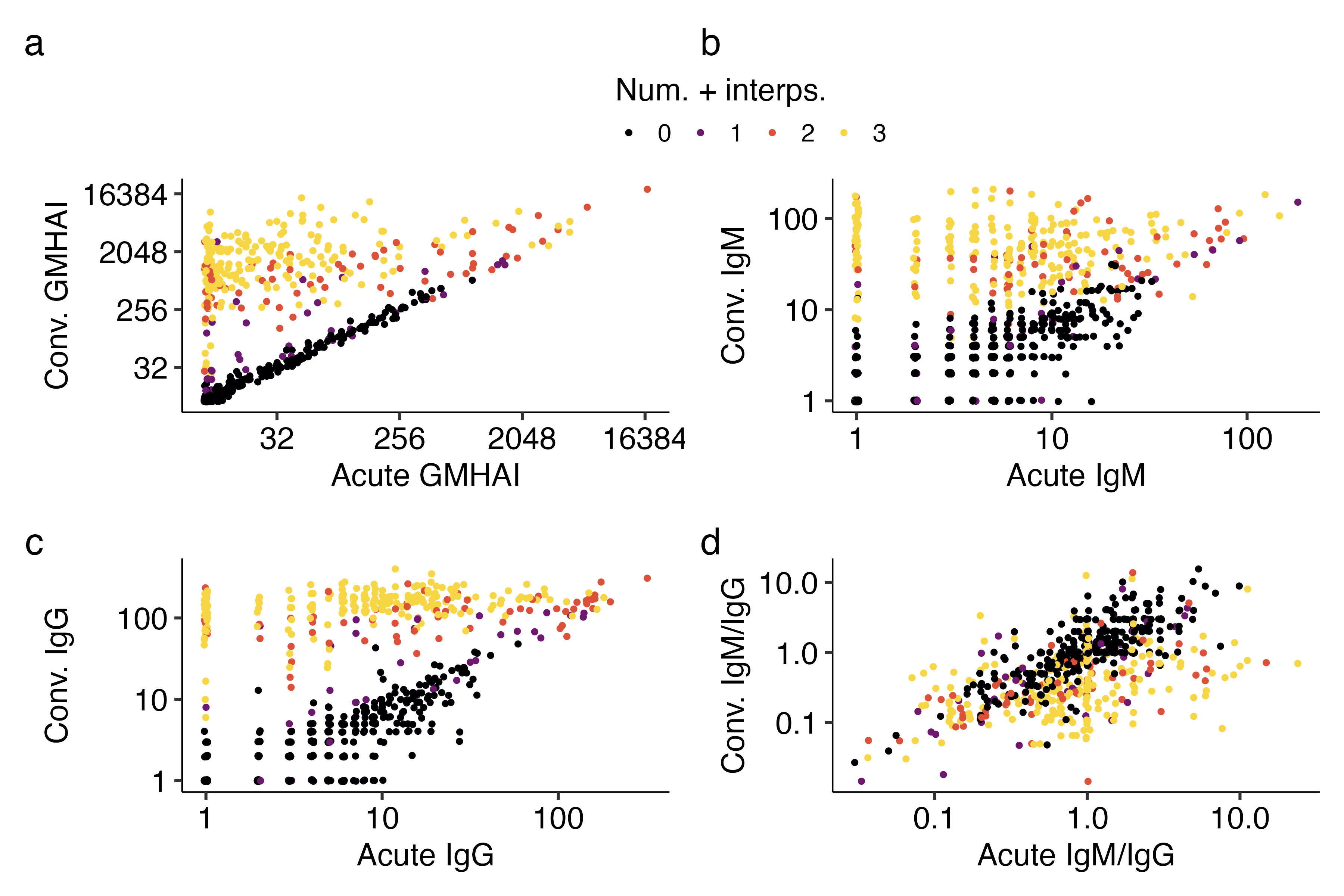

Supplement: S2 Fig — (TIF) [file pcbi.1013708.s008.tif]

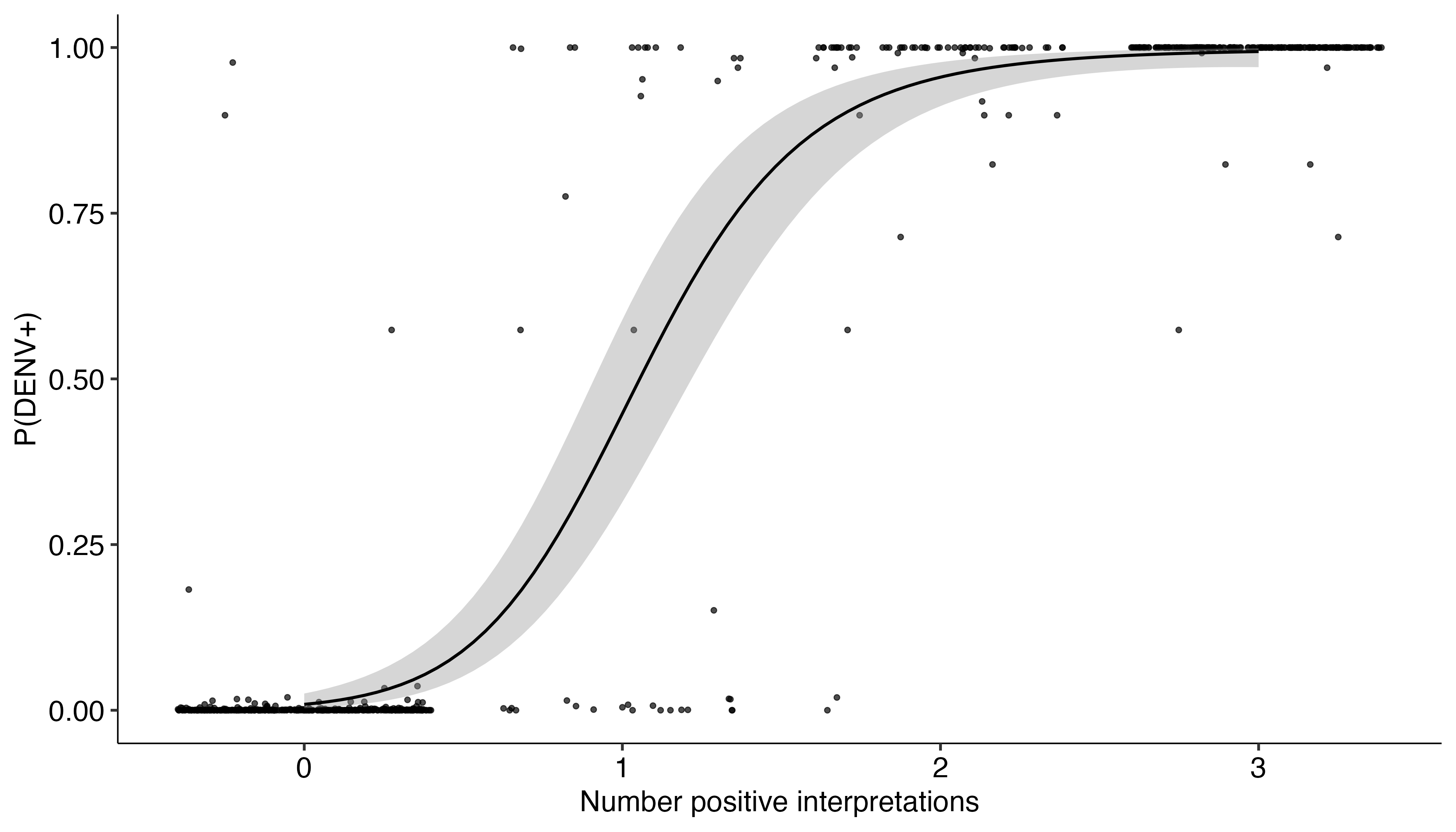

Supplement: S3 Fig — (TIF) [file pcbi.1013708.s009.tif]

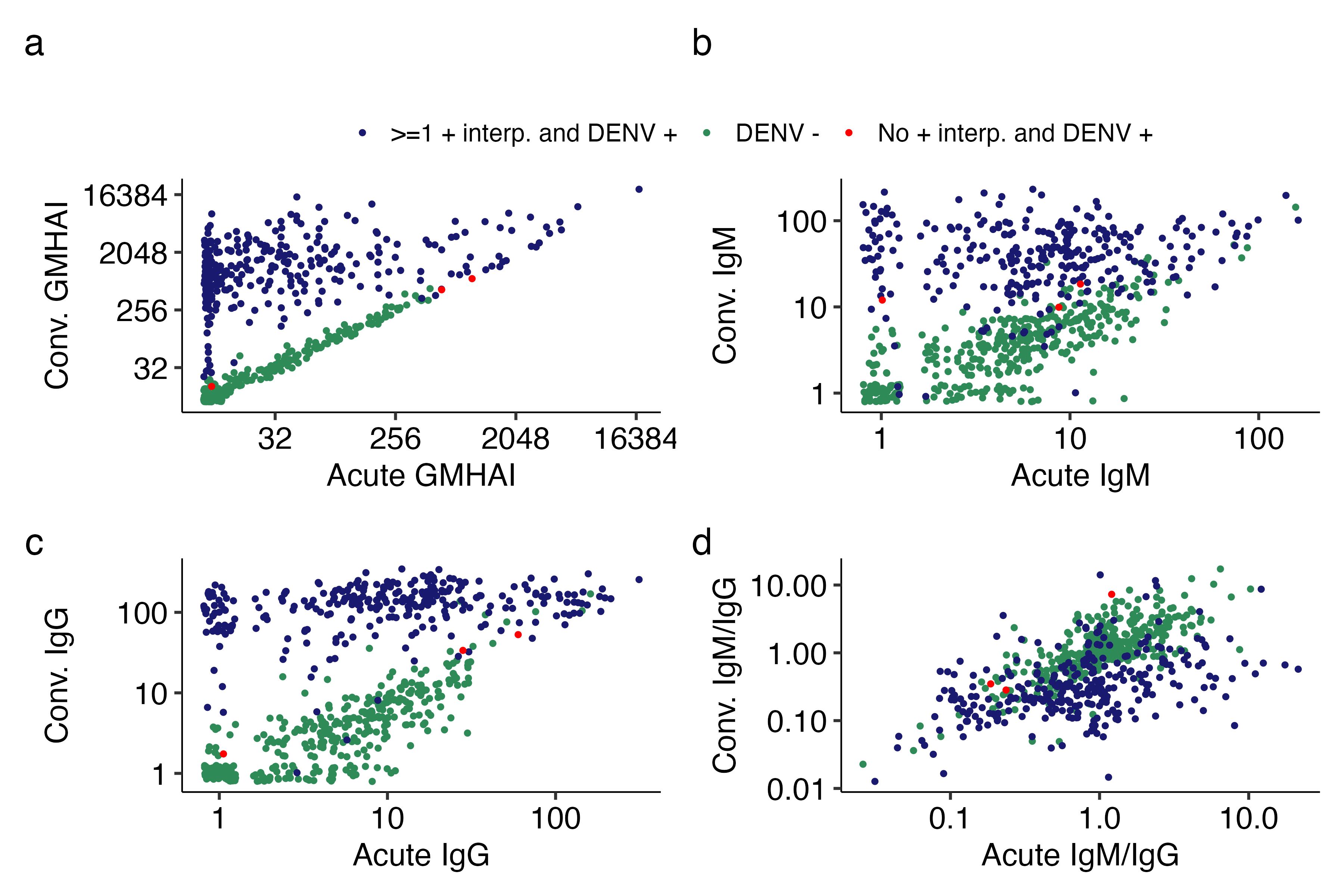

Supplement: S4 Fig — Green points represent no predicted infection while samples classified as an infection are split into two groups, those with no positive serological interpretations (RT-PCR, HAI, and EIA) in red while those with at least one positive serological interpretation are blue. a) Geometric mean titers of a haemagglutination inhibition assay (GM HAI) for all four serotypes of dengue virus. b) Immunoglobulin G (IgG) c) Immunoglobulin M (IgM) d) Ratio of IgM to IgG at both acute and convalescent serum samples. (TIF) [file pcbi.1013708.s010.tif]

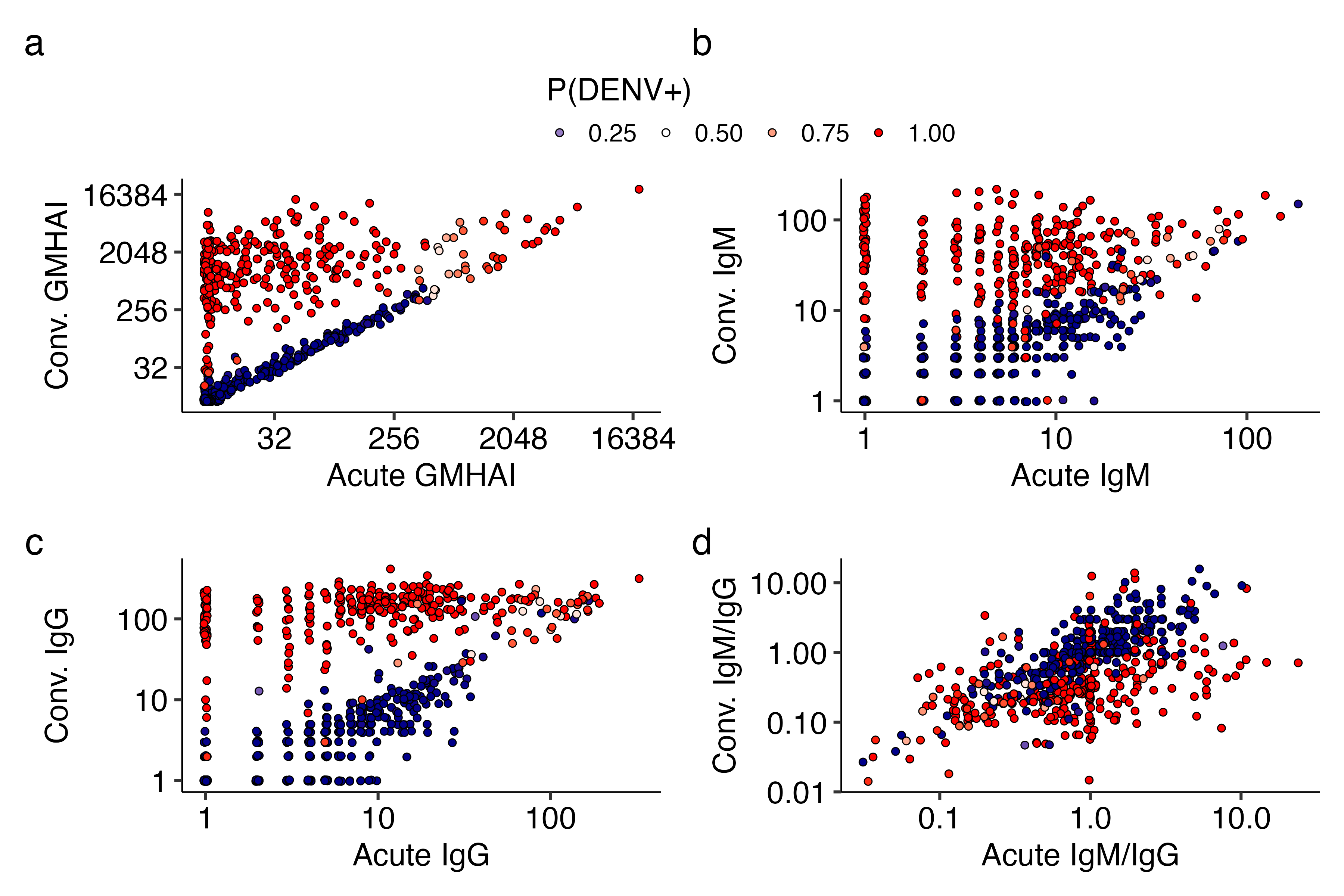

Supplement: S5 Fig — (TIF) [file pcbi.1013708.s011.tif]

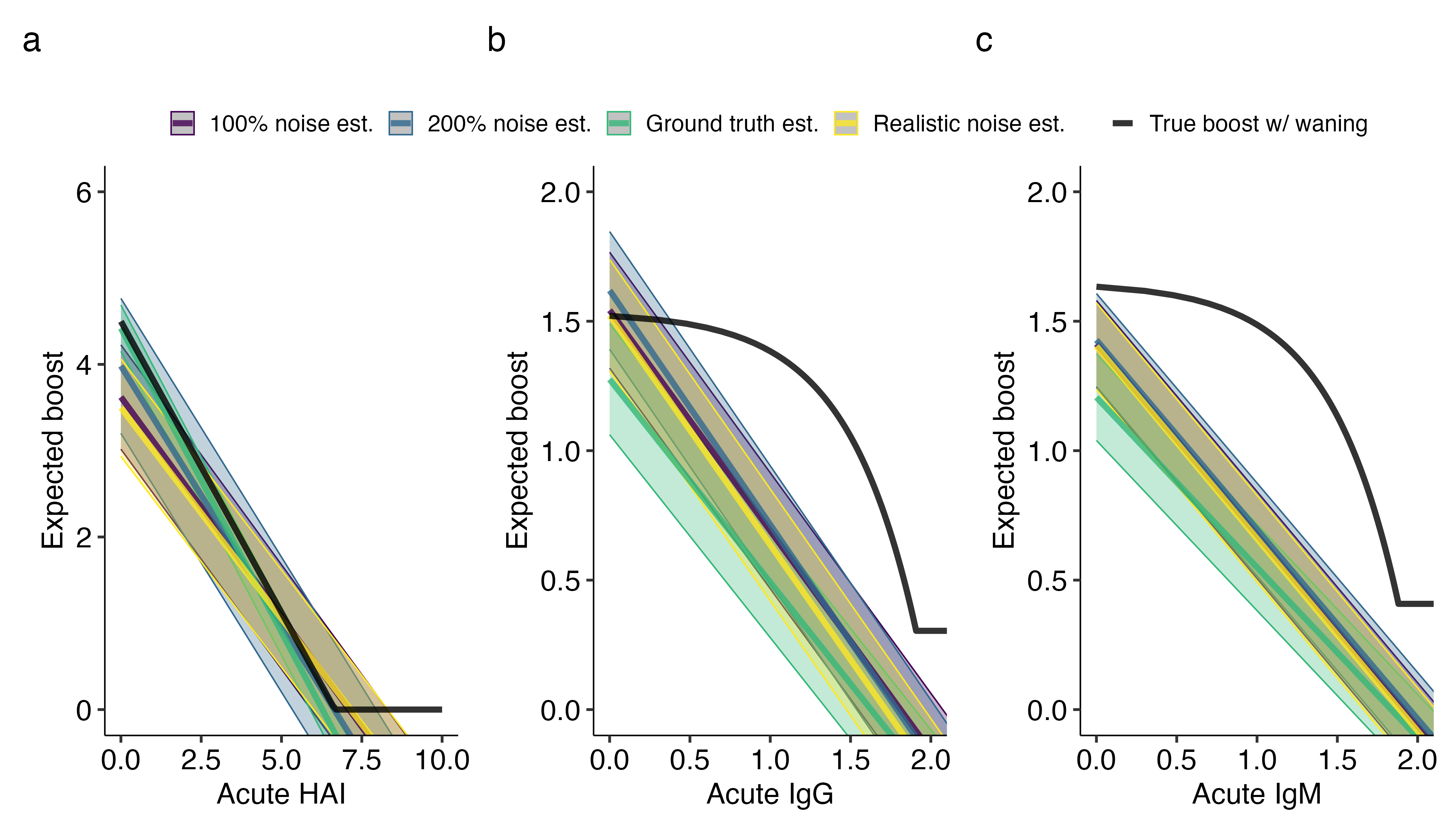

Supplement: S6 Fig — The black solid lines represent the defined functional relationship between acute and convalescent titers after the boost and subsequent waning of titers between the samples. (TIF) [file pcbi.1013708.s013.tif]

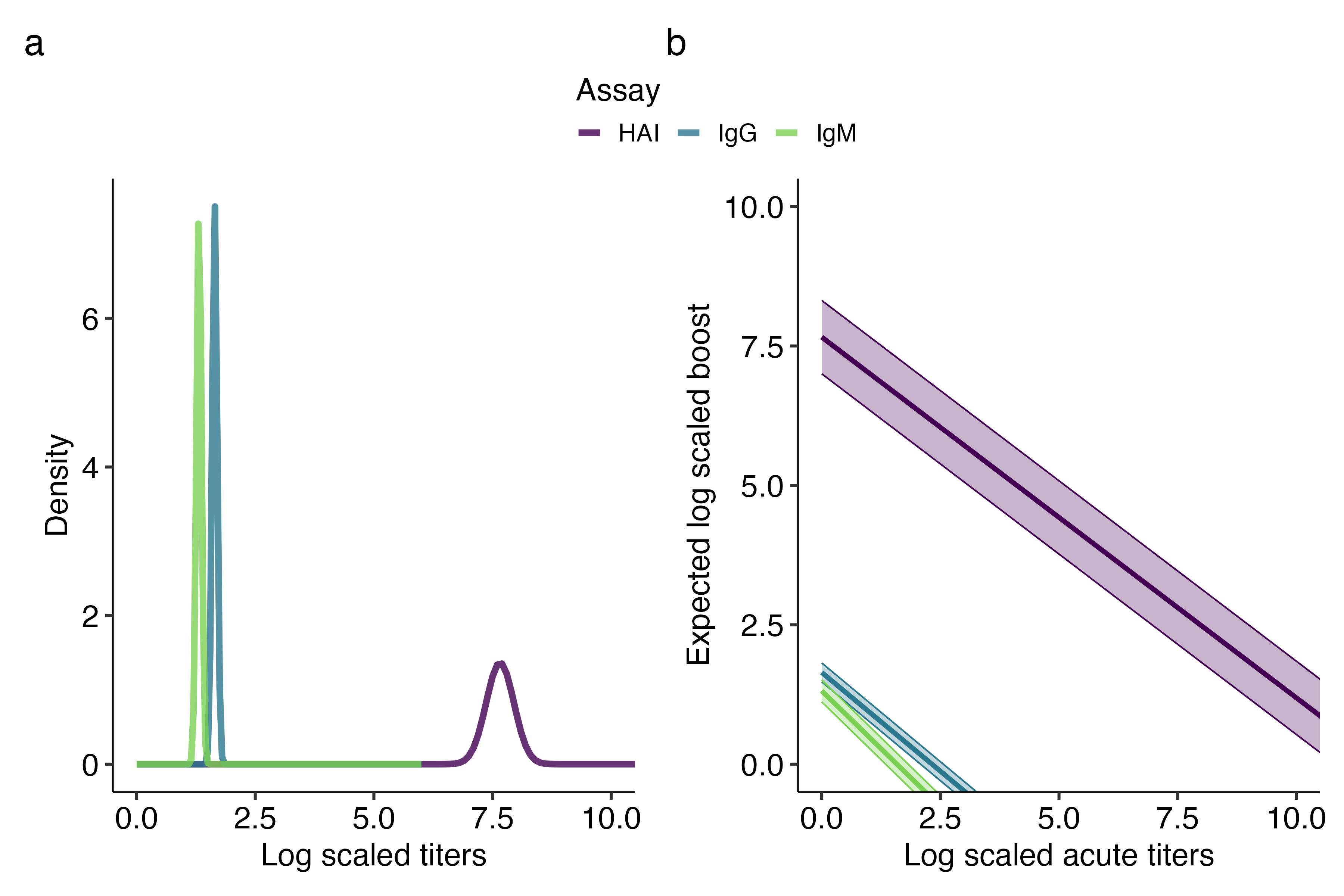

Supplement: S7 Fig — (B) Estimated relationship between titer boost and acute titer for each assay. (PNG) [file pcbi.1013708.s014.png]
