# Supplementary material for: Linking multiple serological assays to infer dengue virus infections from paired samples using mixture models
Source: PLoS Comput Biol. 2025 Nov 25;21(11):e1013708. doi: 10.1371/journal.pcbi.1013708 (PMC12646409; doi:10.1371/journal.pcbi.1013708)
Supplement: S1 Text — (DOCX) [file pcbi.1013708.s004.docx]

**Supplemental**

*Dengue virus nested reverse-transcriptase polymerase chain reaction (Nested RT-PCR)*

The DENV nested RT-PCR was performed following the method previously described by Klungthong et al. 2015. The method comprised two sequential RT-PCR rounds: the 1^st^ round RT-PCR and the subsequent 2^nd^ round nested RT-PCR. For the 1^st^ round RT-PCR, a pair of universal DENV forward and reverse primers were used. This step involved a single RT-PCR procedure, initiated with a RT step at 42°C for 60 minutes, followed by RT-PCR amplification for 35 cycles with the thermocycling conditions of denaturation at 94°C for 30 seconds, annealing at 55°C for 1 minute, and extension at 72°C for 2 minutes. Following the completion of the 1^st^ round RT-PCR, the RT-PCR products were specifically diluted and utilized as the template for the subsequent 2^nd^ round nested RT-PCR. The nested RT-PCR assay employed a mixture of five primers, including the universal forward primer used in the 1st round RT-PCR and four DENV type-specific reverse primers. The 2^nd^ round nested RT-PCR was performed using 25 cycles with the same thermocycling conditions as the 1^st^ round RT-PCR step. After the nested RT-PCR amplification, the RT-PCR products were subjected to agarose gel electrophoresis for analysis. The presence of specific DNA bands on the gel enabled the identification of DENV specimens containing types 1, 2, 3, or 4. Specifically, the detection of a DNA band of 482, 119, 290, and 392 base pairs (bp) indicates DENV-1, DENV-2, DENV-3, and DENV-4, respectively. These DNA bands were compared to the amplified DNA from positive controls, which represent the DENV genome. The composition of the RT-PCR buffer mixture and the primers were described previously [^17^](https://www.zotero.org/google-docs/?TLuVaV).

*Hemagglutination inhibition assay (HAI)*

HAI was carried out using goose erythrocytes as previously described [^45^](https://www.zotero.org/google-docs/?ZYNSjS). In short, sucrose-acetone extracted DENV1 (Hawaii), DENV-2 (NGC), DENV-3 (H87), DENV-4 (H241), and JEV (JaGAr01) antigens from suckling mouse brain have been used as hemagglutinating antigens. Test sera, positive and negative controls were serial 2-fold diluted starting from 1:10 and extending to 1:20,480 and then 250 µL of each dilution was transferred into a v-bottom 96-well plate (Thermo Scientific, US). Then, an equal volume of antigen (8-16 HA units) was added and incubated at 4^o^C overnight before adding 500 µL of goose red blood cells. After incubation at room temperature for 2 hours, the hemagglutination reaction was observed. A non-inhibitory titer of 1:10 is annotated as <10.

*Anti-dengue/JE IgM/IgG enzyme immunoassay*

Anti-DENV/JEV IgM and IgG capture EIA was used in this study and performed in duplicate wells of 96-well flat-bottom microplate. Briefly, microplates were coated with 100 μL/well of 1:1,600 dilution of goat anti-human IgM or IgG (KPL, Gaithersburg, MD) in 0.018 M carbonate buffer (pH 9.0). After overnight incubation at 4°C, the plates were washed with phosphate buffered saline (PBS) (pH 7.4) containing 0.5% Tween 20 (PBS-T). Next, 50 μL/well of 1:100 dilution of test serum, negative control (NC), weak positive control (WPC), and strong positive control (SPC) in PBS were added and incubated overnight at 4°C. After washing with PBS-T, 50 μL/well of sucrose acetone extracted suckling mouse brain DENV (pooled DENV antigen: DENV-1 [Hawaii], DENV-2 [NGC], DENV-3 [H87], and DENV-4 [H241]) and JEV (JaGAr01) antigens were added into DENV (IgM/IgG) and JEV (IgM/IgG) plates, respectively. After incubation for 2 h at room temperature, 30 μL/well of human anti-flavivirus IgG–horseradish peroxidase conjugated was added and incubated for 1 hour at 37°C. After washing with PBS-T, 100 μL/well of TMB substrate (KPL, Gaithersburg, MD) was added and incubated for 10–30 min. The reaction was stopped by adding 50 μL/well of 0.2 M sulfuric acid. The absorbance (optical density [OD]) was measured at a wavelength of 450 nm (SoftMax Pro Software, Molecular Devices, San Jose, CA). A valid assay should provide OD values at < 0.100, 0.400–0.600, and > 0.600 for NC, WPC, and SPC, respectively. EIA units of tested serum are equal to 100 × [(ODTest − ODNC)/(ODWPC − ODNC)].

*Classification as primary or post-primary DENV infection*

Acute primary DENV infection was defined by a 4-fold rise and HAI titers at or below 1,280. Acute post-primary DENV infection was defined by a 4-fold rise and HAI titers at or higher than 2,560. Recent DENV infections were defined by HAI titers at or higher than 2,560 at the acute sampling event. These are infections estimated to have occurred more than one week or less than two months ago, given the high titers measured at the acute visit. For EIA interpretations units of IgM ≥ 40 were used as a positive cutoff value. Evidence of DENV infection was classified by a ratio of DENV to JEV IgM ≥ 1.0, and JEV infection when the ratio was < 1.0. Acute primary DENV infection was interpreted when the ratio of DENV IgM/DENV IgG was ≥ 1.8, and acute post-primary DENV infection was considered when the ratio was < 1.8.

*Models incorporating baseline acute titers*

This extension incorporated the baseline acute titers of an individual prior to the acute and convalescent samples. Here we replaced all acute samples with the most recent serological datapoint prior to symptom onset. This was developed to remove some of the issues with high acute titers during the acute visit under the hypothesis that the previous serological sample could better reflect antibody levels prior to infection. However, we had similar issues with some serological samples having high initial titers, likely having experienced a recent infection.

*Models incorporating timing of samples*

This extension was developed to leverage the timing of the acute samples and difference in time between the acute and convalescent sample as the timing of measurements will determine where along the antibody kinetic trajectory we sample from. We developed alternative univariate and multivariate mixture models to Equation 1 and 2 by incorporating the time since symptom onset as an additional covariate that could impact the resulting convalescent titers as follows:

$f\left( y_{i}^{C} \right) =\theta_{0}Normal\left( y_{i}^{C}|y_{i}^{A},\sigma_{0} \right) +\theta_{1}Normal\left( y_{i}^{C}|{\mu+\beta_{1} y}_{i}^{A}{+\beta_{2} t}_{i}^{s},\sigma_{1} \right)$, (3)

Where $t_{i}^{s}$ is the time since symptom onset for the acute sample and $\beta_{1}$ and $\beta_{1}$ are the estimated parameters for how acute titers and time since symptom onset impact convalescent titers. A similar extension for the multivariate case (Equation 2) can be performed as well. Neither approach led to better fits, with the latter approach making model fit significantly more difficult due to the increased complexity.

*Relative observational noise estimation*

For the simulated data three different levels of observational noise were chosen for each assay. To quantify the relative magnitude of observational noise we calculated the standard deviation of the difference in titers between acute and convalescent samples in individuals without recent infections. We chose this as a reference group since it is expected that any change in titers between these two time points will be due to biological variability or assay noise. While there might also be some antibody decay, it is likely to be minimal due to the temporal proximity of the samples. To ensure comparability of the standard deviations, we divided them by the total assay range.

$ObsNoise= sd({|x}_{acute}-x_{convalescent}|)/(max(x_{convalescent}) - min(x_{acute}))$ where $x_{acute}$ and $x_{convalescent}$ are the collection of acute and convalescent samples from uninfected individuals. We quantify this metric for each combination of assay and noise/dataset and present the results in S2 Table.
