# Supplementary material for: Linking multiple serological assays to infer dengue virus infections from paired samples using mixture models
Source: PLoS Comput Biol. 2025 Nov 25;21(11):e1013708. doi: 10.1371/journal.pcbi.1013708 (PMC12646409; doi:10.1371/journal.pcbi.1013708)
Supplement: Attachment — Submitted filename: Plos_comp_review_May25.docx [file pcbi.1013708.s015.docx]

Please find below my review for the manuscript: “Linking multiple serological assays to infer dengue virus infections from paired samples using mixture models”, for which my recommendation is ‘accept with minor revisions’.

I’ve split my comments by section so the authors can match them to their text. The majority of comments are suggestions for only minor revisions, alongside comments about the need for better signposting to descriptions for each model, increasing those descriptions especially for models applied to the Thai data, and moving the model descriptions to the methods section.

The work is impressive and I enjoyed reading this paper. I hope that the authors find these review comments helpful.

Summary

Hamins-Puértolas *et al*. report work applying mixture models fitted to serological data from multiple sources to infer dengue infections. The work is original, innovative, and of high importance to the field given the methodological insights it describes. Code and data have been made publicly available through a GitHub repository.

Review comments

Introduction

- Cut off and cutoff both used in introduction (and cut-off in methods). One should be used throughout for consistency.
- Fourfold and two fold inconsistent formatting (and 4-fold, four-fold in the methods sections)

Methods

- ‘(GM HAI or IgG)’ is IgM missing here?
- In the section ‘Mixture models for serological data from a single acute or convalescent sample ‘, when mean and variance are introduced , the super and subscripts need defining.
- Double full stop at end of 3^rd^ methods paragraph.
- ‘We simulated the serological response to three serological assays for all individuals (Figure 2)’ . I would state here that the 3 mean IgG, IgM, and HAI rather than later in the paragraph. And how big is your example population in the simulated data? I see now that it is 500 from the results section, please add that to this methods section too.
- I see that the age range of the two cohort studies is in Table 1, but I would add that children means 0-18 year olds in the main text about KPS1.
- How were the 494 households chosen/ enrolled in the KFCS? Is there a reference to the study that can be used here? If not, then I would recommend including a little more background information on the sampling and design in the main or supplementary text.
- Et al in the laboratory methods section should be in italics.
- ‘by a ratio of DENV IgM/JEV IgM ≥ 1.0’ in this sentence the second IgM is potentially an error.

Results

- ‘F1 score ranging from 98.0-93.9% as observational noise was increased’ Consider switching and rewording the sentence because it looks unusual to have ranges decrease.
- ‘…leading to seven total models’ I would recommend listing out in the text e.g. Model 1 = XXX, Model 2 = XXX, and having a table describing the model variations. I see now that this is covered by Table 2 – I recommend referencing Table 2 after the sentence (and Table S5 for models 10-15). Naming and description of all the models should come in the methods section.
- For the 15 models run for the Thai data, more description of the extra model variations (8-15) which are not run on the simulated data is needed; how do they differ to 1-7?
- ‘ranging from small to large amounts of observational noise’ Some quantification of small and large would be good.
- Gold standard and gold-standard are used inconsistently throughout
- ‘Model 9 (GM HAI, IgG, IgM, and an acute GM HAI univariate mixture model, Figure 3) consistently performed well across both gold-standard definitions of infection (Table 2), where it had high sensitivity (96.1% and 85.2%) and specificity (86.2% and 97.7%)’. It could be clearer which value refers to which infection definition here.
- ‘The remaining lower certainty paired samples were more likely to come from disagreeing interpretations (ie. one or two positive serological interpretations) than not (Figure S2).’ A quantitative representation of ‘more likely’ in this part of the text would be useful.

Discussion

- Minor suggestion here to state again the time window for ‘acute or recent’ infections.
- ‘Previous work has demonstrated that a simple combination of classification results can lead to increased sensitivity, but at times can at the cost of reduced specificity’. There is potentially a missing word somewhere in this sentence.
- ‘One major limitation is that the paired model is at times not sufficient and two independent models (recent infection and paired model) had to be combined to explain some paired samples with high acute titers.’ How many is ‘some’?
- ‘for epidemiologic and clinical studies’, I think this should this be epidemiological.
- ‘these methods are highly transportable’, should this be transferable?
- You’ve highlighted how your methods are increasingly applicable as multiplex assays become more common in epidemiological studies. I would recommend highlighting this strength / commenting on multiplex assays in the introduction section more too.

Figures and Tables

- Would the information in Table 1 be clearer as a multipaneled figure?
- In Table 1 caption, it would be useful to state that the ‘data analysed’ is the Kamphaeng Phet cohort studies KPS1 and KFCS so it can stand alone without the reader looking in the text. Also definitions for the acronyms HAI and EIA. I found the placement of the Table odd, given it isn’t referenced until much later in the manuscript.
- Same as my comment for Table 1 – would the information in Table 2 be better visualised as a multipanel bar figure? For example a panel for each metric, coloured by model. It’s quite difficult to compare between all the models in the table.
- Table S3. What does lowest, additional, and highest noise mean? I think you need to quantify the differing amounts in the table caption.
