# Supplementary material for: Linking multiple serological assays to infer dengue virus infections from paired samples using mixture models
Source: PLoS Comput Biol. 2025 Nov 25;21(11):e1013708. doi: 10.1371/journal.pcbi.1013708 (PMC12646409; doi:10.1371/journal.pcbi.1013708)
Supplement: Attachment — Submitted filename: Response to Reviewers - PLOS CB 2025 Revise and resubmit.docx [file pcbi.1013708.s016.docx]

Dear Drs. Kraemer and Kouyos,

Thank you for the opportunity to submit a revised draft of the manuscript “Linking multiple serological assays to infer dengue virus infections from paired samples using mixture models” for publication in PLOS Computational Biology. We appreciate the time and effort of the editorial team and reviewers. We have incorporated suggestions made by the reviewers and have highlighted these changes in red below.

**Reviewer #1:** Please find below my review for the manuscript: “Linking multiple serological assays to infer dengue virus infections from paired samples using mixture models”, for which my recommendation is ‘accept with minor revisions’.

I’ve split my comments by section so the authors can match them to their text. The majority of comments are suggestions for only minor revisions, alongside comments about the need for better signposting to descriptions for each model, increasing those descriptions especially for models applied to the Thai data, and moving the model descriptions to the methods section.

The work is impressive and I enjoyed reading this paper. I hope that the authors find these review comments helpful.

Summary

Hamins-Puértolas *et al*. report work applying mixture models fitted to serological data from multiple sources to infer dengue infections. The work is original, innovative, and of high importance to the field given the methodological insights it describes. Code and data have been made publicly available through a GitHub repository.

Review comments

Introduction

1. Cut off and cutoff both used in introduction (and cut-off in methods). One should be used throughout for consistency.
   *Thank you for noting this, both edits were added for consistency.*
2. Fourfold and two fold inconsistent formatting (and 4-fold, four-fold in the methods sections)
   *Thank you for noting this, both edits were added for consistency.*

Methods

3. ‘(GM HAI or IgG)’ is IgM missing here?
   *We have updated the referenced sentence in the Methods section to include IgM. It now reads:
   “We call these the acute or convalescent (GM HAI, IgG, or IgM) univariate mixture model hereafter.”*
4. In the section ‘Mixture models for serological data from a single acute or convalescent sample ‘, when mean and variance are introduced , the super and subscripts need defining.
   *We have removed the “R” superscripts and defined the m to be representative for each mixture. It now reads:
   “This is done using a single univariate mixture model with M total mixtures, each of which has a mean and variance of* $\mu_{m}$ *and* $\sigma_{m}$*respectively for each mixture m.”*
5. Double full stop at end of 3^rd^ methods paragraph.
   *We have removed the double full stop here.*
6. ‘We simulated the serological response to three serological assays for all individuals (Figure 2)’ . I would state here that the 3 mean IgG, IgM, and HAI rather than later in the paragraph. And how big is your example population in the simulated data? I see now that it is 500 from the results section, please add that to this methods section too.
   *We have updated the Simulation of serological data subsection of the methods to reflect this number. In particular we have written:
   We simulated the serological response to three serological assays* ***(IgG, IgM, and GM HAI)*** *for* ***500*** *individuals (Figure 2).*
7. I see that the age range of the two cohort studies is in Table 1, but I would add that children means 0-18 year olds in the main text about KPS1.
   *KPS1 consists of children between seven and 15 years of age while KFCS contains individuals from 0 to 91 years of age. We have updated the Methods section to mention this fact. In particular, we have updated the Cohort data description to say:
   The first cohort study, the Kamphaeng Phet Study (KPS1), recruited 3,647 children* ***(aged seven to 15)*** *from 12 different primary schools and ran from 1998 to 2002.*
8. How were the 494 households chosen/ enrolled in the KFCS? Is there a reference to the study that can be used here? If not, then I would recommend including a little more background information on the sampling and design in the main or supplementary text.
   *Enrollment in the cohort is described in detail in Anderson et al., 2020. In summary, eligible (residents of Kamphaeng Phet, Thailand, no chronic illness, immunodeficiency, or recent immunosuppressive therapy, and at least four total household residents including a grandparent and child in addition to the pregnant mother and their newborn) pregnant women in their third trimester were identified. These mothers and their households were consented and enrolled such that upon birth of the newborn all individuals underwent yearly serological sampling. We have included a reference to the study in section Cohort data description of the manuscript.*
9. Et al in the laboratory methods section should be in italics.
   *We have corrected this.*
10. ‘by a ratio of DENV IgM/JEV IgM ≥ 1.0’ in this sentence the second IgM is potentially an error. *We have updated the text as follows:
    “by a ratio of DENV to JEV IgM ≥ 1.0”*

Results

11. ‘F1 score ranging from 98.0-93.9% as observational noise was increased’ Consider switching and rewording the sentence because it looks unusual to have ranges decrease.
    *We have changed the phrasing to make the scores increase by noting that this occurs when observational noise decreases. We updated this sentence found in the second paragraph of the Results to read:
    “The model incorporating all three assays had an F1 score* ***increasing*** *from* ***93.9-98.0%*** *as observational noise was* ***decreased.****”*
12. ‘…leading to seven total models’ I would recommend listing out in the text e.g. Model 1 = XXX, Model 2 = XXX, and having a table describing the model variations. I see now that this is covered by Table 2 – I recommend referencing Table 2 after the sentence (and Table S5 for models 10-15). Naming and description of all the models should come in the methods section.

*See response to comment 13*

13. For the 15 models run for the Thai data, more description of the extra model variations (8-15) which are not run on the simulated data is needed; how do they differ to 1-7?
    *For both comments 12 and 13 we have added additional descriptions to the Model fitting subsection in the Methods for the Models. This reads as follows:*

*“All models were implemented in stan. We fit 15 total models: Models 1-9 incorporated data from each combination of the three assays at both acute and convalescent time points (Table 2) while Models 10-15 fit univariate mixture models to one assay and time point (acute or convalescent) at a time (Table S5). Models 7 and 9 combined multivariate and univariate mixture models to account for high acute titers.“*

14. ‘ranging from small to large amounts of observational noise’ Some quantification of small and large would be good.
    *We thank the reviewer for pointing this out. We have included quantification of the levels of noise as described below. As shown in Table S2, the “high noise” was equal or higher than the observed variability of the cohort data used.*

    *We have added the following to the Simulation of serological data section of the main text to provide more information on a direct quantification of the observational noise implemented in the simulated data:*

*The relative observational noise used is presented in Table S2 allowing for direct comparison across assays as well as across the different datasets (simulated and cohort data).

We have also added the following subsection to the supplemental to quantify the different levels of noise:*

*Relative observational noise estimation*

*For the simulated data three different levels of observational noise were chosen for each assay. To quantify the relative magnitude of observational noise we calculated the standard deviation of the difference in titers between acute and convalescent samples in individuals without recent infections. We chose this as a reference group since it is expected that any change in titers between these two time points will be due to biological variability or assay noise. While there might also be some antibody decay, it is likely to be minimal due to the temporal proximity of the samples. To ensure comparability of the standard deviations, we divided them by the total assay range.*

*We can write this metric as:*

$Ob{sNoise}= sd({|x}_{acute}-x_{convalescent}|)/(max(x_{convalescent}) - min(x_{acute}))$ *where* $x_{acute}$ *and* $x_{convalescent}$ *are the collection of acute and convalescent samples from uninfected individuals. We quantify this metric for each combination of assay and noise/dataset and present the results in Table S2.*

***Table 1.*** *Relative observational noise by assay and dataset. Methodology outlined in the Relative observational noise estimation section of the Supplemental information.*

|  | ***Assay*** | | |
| --- | --- | --- | --- |
| ***Dataset*** | ***GM HAI*** | ***IgM*** | ***IgG*** |
| *Lowest noise* | *0.015* | *0.069* | *0.114* |
| *Additional noise* | *0.037* | *0.092* | *0.141* |
| *Highest noise* | *0.071* | *0.122* | *0.195* |
| *Cohort data* | *0.018* | *0.119* | *0.107* |

15. Gold standard and gold-standard are used inconsistently throughout
    *We thank the reviewer for this comment. We have gone ahead and ensured that gold-standard was used as an adjective and gold standard as a noun in the text.*
16. ‘Model 9 (GM HAI, IgG, IgM, and an acute GM HAI univariate mixture model, Figure 3) consistently performed well across both gold-standard definitions of infection (Table 2), where it had high sensitivity (96.1% and 85.2%) and specificity (86.2% and 97.7%)’. It could be clearer which value refers to which infection definition here.
    *We thank the reviewer for noting this. We have updated this sentence found in the third paragraph of the Application of the model to paired serological data from Thailand subsection of the Results to read:
    “Model 9 (GM HAI, IgG, IgM, and an acute GM HAI univariate mixture model, Figure 3) consistently performed well across both* ***RT-PCR and combined serological*** *definitions of infection (Table 2)...”*
17. ‘The remaining lower certainty paired samples were more likely to come from disagreeing interpretations (ie. one or two positive serological interpretations) than not (Figure S2).’ A quantitative representation of ‘more likely’ in this part of the text would be useful.
    *We have made these edits in response #14 above.*

Discussion

18. Minor suggestion here to state again the time window for ‘acute or recent’ infections.
    *Acute infections are defined to be detectable within seven days of symptom onset with convalescent titers often rising and leading to positive serological interpretation. Individuals who present with very high titers at the acute visit are classified as having a recent infection. It is likely that these infections have occurred more than a week prior to sampling but within the previous two months (Salje et al., 2018). We have updated the manuscript to note this. In particular we have written in the Classification as primary or post-primary DENV infection section in reference to what a recent infection is:*

*“These are infections estimated to have occurred more than one week or less than two months ago, given the high titers measured at the acute visit.”*

*In this same section we changed all references of “primary DENV infection” and “post-primary DENV infection” to be read as “acute primary DENV infection” and “acute post-primary DENV infection” for consistency.*

19. ‘Previous work has demonstrated that a simple combination of classification results can lead to increased sensitivity, but at times can at the cost of reduced specificity’. There is potentially a missing word somewhere in this sentence.
    *This error has been fixed to read:
    “Previous work has demonstrated that a simple combination of classification results can lead to increased sensitivity,* ***often*** *at the cost of reduced specificity"*
20. ‘One major limitation is that the paired model is at times not sufficient and two independent models (recent infection and paired model) had to be combined to explain some paired samples with high acute titers.’ How many is ‘some’?
    *We have removed ‘some’ due to its ambiguity. ‘Some’ was originally used to describe the less than 5% of paired samples that follow this pattern.*
21. ‘for epidemiologic and clinical studies’, I think this should this be epidemiological.
    *This error has been fixed.*
22. ‘these methods are highly transportable’, should this be transferable?
    *This error has been fixed.*
23. You’ve highlighted how your methods are increasingly applicable as multiplex assays become more common in epidemiological studies. I would recommend highlighting this strength / commenting on multiplex assays in the introduction section more too.
    *We have added the following sentence to the introduction to further highlight the strength of this approach as multiplex serological panels continue to advance:
    “Recent development of multiplex serological panels only further demonstrates the need for methods that can leverage multiple data streams.” to the introduction to highlight the applicability of this approach on multiplex assays.*

Figures and Tables

24. Would the information in Table 1 be clearer as a multipaneled figure?

*We understand the questions regarding visualization for Tables 1 and 2, however we believe that this would not fix the underlying issue that there are many models the reader must keep track of. Instead, we believe that the updated methods section outlining the differences between the models will help readers differentiate between each of the models.*

25. In Table 1 caption, it would be useful to state that the ‘data analysed’ is the Kamphaeng Phet cohort studies KPS1 and KFCS so it can stand alone without the reader looking in the text. Also definitions for the acronyms HAI and EIA. I found the placement of the Table odd, given it isn’t referenced until much later in the manuscript.
    *We have gone ahead and updated the Table 1 caption to represent where the analysed data comes from. In addition we added definitions for HAI and EIA as well as adding a reference to Table 1 in the section Cohort data description, which was previously missing.*
26. Same as my comment for Table 1 – would the information in Table 2 be better visualised as a multipanel bar figure? For example a panel for each metric, coloured by model. It’s quite difficult to compare between all the models in the table.
    *Response to this can be found above in response #24.*
27. Table S3. What does lowest, additional, and highest noise mean? I think you need to quantify the differing amounts in the table caption.
    *Response to this can be found above in response #14.*

**Reviewer #2:** The authors present a method to jointly analyse multiple serological assays from paired samples. They use mixture models, the application of which is layed out in the manuscript. They show that the use of additional assays improves the accuracy of infection detection. The manuscript is well written, the concepts are well explained, and the approach is well chosen and validated. I only have some minor comments that would help increase the clarity of the manuscript and future use of the methods proposed.

28. The authors discuss some extensions to their framework, including adding baseline titers and the time since symptom onset as covariates. It would be helpful for future applications if the authors would describe the methodology used in these additional steps more explicitly.
    *We have added the following section to supplemental to outline these additional methods.*

*“Models incorporating baseline acute titers*

*This extension incorporated the baseline acute titers of an individual prior to the acute and convalescent samples. Here we replaced all acute samples with the most recent serological datapoint prior to symptom onset. This was developed to remove some of the issues with high acute titers during the acute visit under the hypothesis that the previous serological sample could better reflect antibody levels prior to infection. However, we had similar issues with some serological samples having high initial titers, likely having experienced a recent infection.*

*Models incorporating timing of samples*

*This extension was developed to leverage the timing of the acute samples and difference in time between the acute and convalescent sample as the timing of measurements will determine where along the antibody kinetic trajectory we sample from. We developed alternative univariate and multivariate mixture models to Equation 1 and 2 by incorporating the time since symptom onset as an additional covariate that could impact the resulting convalescent titers as follows:*

$f\left( y_{i}^{C} \right) =\theta_{0}Normal\left( y_{i}^{C}|y_{i}^{A},\sigma_{0} \right) + \theta_{1}Normal\left( y_{i}^{C}|{\mu+\beta_{1} y}_{i}^{A}{+\beta_{2} t}_{i}^{s}{,\sigma_{1}} \right)$*, (3)*

*Where* $t_{i}^{s}$  *is the time since symptom onset for the acute sample and* $\beta_{1}$ and $\beta_{1}$ *are the estimated parameters for how acute titers and time since symptom onset impact convalescent titers. A similar extension for the multivariate case (Equation 2) can be performed as well. Neither approach led to better fits, with the latter approach making model fit significantly more difficult due to the increased complexity.”*

29. Similarly, the simulation of the synthetic data is performed using the serosim package. It would be helpful if, in addition to the parameter used (table S1) the model used for the generation of the data was given (in main of SM). In particular, it was not clear to me how the immune history was modelled.

*Immune history was defined by simulating a long period with a low force of infection, where individuals were infected, had antibody boosts and subsequent decays. This was simulated such that when a short period with a high force of infection was run, individuals with a wide range antibody profiles (i.e., individuals who had experienced different numbers of prior infections) were infected. The antibody levels of an exposed individual also defined the probability that they would be infected through a logistic transformation.*

*We have added some information to further elaborate on this simulation approach in the Simulation of serological data subsection of the Methods. We incorporated more information on how exposure, infection, and subsequent antibody kinetics were defined. The updated section reads as follows:*

*“Simulation of serological data*

*To assess the performance of our proposed model we first used simulated data generated using serosim, a flexible R package capable of simulating serological data which we ran in R version 4.3.3 39. We simulated the serological response to three serological assays (IgG, IgM, and GM HAI) for 500 individuals (Figure 2). This was done by defining exposure, infection given an exposure, and subsequent antibody kinetics post infection. The population as a whole underwent an extended period of low force of infection to the pathogen during which individuals could be infected, followed by a short period of high force of infection that infected approximately half of the population. After every successful infection an individual’s antibody responses would undergo a boost in titers for all three assays followed by antibody decay. Upper and lower bounds of detection for each assay were pre-defined, and the relationship between an individual’s initial titers and the expected boost in titers was also defined such that individuals with high titers experienced a smaller boost in titers. The chosen parameters for all antibody kinetics can be found in Table S1. Note that this approach assumes an immediate boost post infection followed by a decay in titers, and does not consider differences in the timing of boosts that may exist between primary and post-primary infections.*

*Simulated individuals could experience up to four sequential infections. Each individual’s antibody profile, reflective of the past infection history and measured through antibody levels, impacted their probability of infection during a subsequent exposure window through a logistic transformation reflecting immune-mediated protection. Individuals with a simulated log2 GMHAI titer greater than 8 had a 50% chance of getting infected as compared to those with no titers. To generate the dataset for analysis, antibody titers consistent with an acute and convalescent time point were extracted from the simulated data. Three different amounts of observational noise were added to the data to quantify how measurement error impacts model performance. The relative observational noise used is presented in Table S2 allowing for direct comparison across assays as well as across the different datasets (simulated and cohort data). The code we utilized for these simulations can be found in the github repository github.com/marcohamins/linking-multiple-sero-assays.”*

30. Lastly, the model fitting approach is very briefly described. What algorithm was used here? This part would benefit from a bit more details.

*We have clarified that we implemented our model in a Bayesian framework using stan in the Model fitting subsection of the Methods section. Stan (as applied in Rstudio using Cmdstanr) implements a No-U-Turn Sampler, an adaptive variant of Hamiltonian Monte Carlo, for posterior sampling.*

**References:**

1. Anderson, Kathryn B., et al. "An innovative, prospective, hybrid cohort-cluster study design to characterize dengue virus transmission in multigenerational households in Kamphaeng Phet, Thailand." American journal of epidemiology 189.7 (2020): 648-659.
2. Salje, Henrik, et al. "Reconstruction of antibody dynamics and infection histories to evaluate dengue risk." Nature 557.7707 (2018): 719-723.
